# Elucidating the impact of *in vitro* cultivation on *Nicotiana tabacum* metabolism through combined *in silico* modeling and multiomics analysis

**DOI:** 10.3389/fpls.2023.1281348

**Published:** 2023-11-03

**Authors:** Jing Yu, Xiaowei Wang, Qianqian Yuan, Jiaxin Shi, Jingyi Cai, Zhichao Li, Hongwu Ma

**Affiliations:** ^1^ National Technology Innovation Center of Synthetic Biology, Tianjin, China; ^2^ Biodesign Center, Key Laboratory of Systems Microbial Biotechnology, Tianjin Institute of Industrial Biotechnology, Chinese Academy of Sciences, Tianjin, China; ^3^ Key Laboratory of Systems Microbial Biotechnology, Tianjin Institute of Industrial Biotechnology, Chinese Academy of Sciences, Tianjin, China

**Keywords:** genome-scale metabolic model, tobacco, flux balance analysis, *in vitro* cultivation, multiomics analysis

## Abstract

The systematical characterization and understanding of the metabolic behaviors are the basis of the efficient plant metabolic engineering and synthetic biology. Genome-scale metabolic networks (GSMNs) are indispensable tools for the comprehensive characterization of overall metabolic profile. Here we first constructed a GSMN of tobacco, which is one of the most widely used plant chassis, and then combined the tobacco GSMN and multiomics analysis to systematically elucidate the impact of *in-vitro* cultivation on the tobacco metabolic network. *In-vitro* cultivation is a widely used technique for plant cultivation, not only in the field of basic research but also for the rapid propagation of valuable horticultural and pharmaceutical plants. However, the systemic effects of *in-vitro* cultivation on overall plant metabolism could easily be overlooked and are still poorly understood. We found that *in-vitro* tobacco showed slower growth, less biomass and suppressed photosynthesis than soil-grown tobacco. Many changes of metabolites and metabolic pathways between *in-vitro* and soil-grown tobacco plants were identified, which notably revealed a significant increase of the amino acids content under *in-vitro* condition. The *in silico* investigation showed that *in-vitro* tobacco downregulated photosynthesis and primary carbon metabolism, while significantly upregulated the GS/GOGAT cycle, as well as producing more energy and less NADH/NADPH to acclimate *in-vitro* growth demands. Altogether, the combination of experimental and *in silico* analyses offers an unprecedented view of tobacco metabolism, with valuable insights into the impact of *in-vitro* cultivation, enabling more efficient utilization of *in-vitro* techniques for plant propagation and metabolic engineering.

## Introduction

Plant metabolic characterization and plant engineering are usually incomprehensive and inefficient, partly because of insufficient information on metabolic flux distributions and poor understanding of metabolic behaviors at the network level (Arya et al., 2020; Lynch et al., 2021). To systematically and globally characterize the metabolic behaviors of plants, it is necessary to reconstruct a genome-scale metabolic networks (GSMNs) that incorporate comprehensive catalytic transformations., which have been utilized to elucidate cellular metabolism and guide metabolic engineering to overproduce desired compounds (Chatsurachai et al., 2012; Kim et al., 2015a; Zhang et al., 2017). In the last decade, many efforts have been made to construct GSMNs for the important plant species, such as *Arabidopsis* (Cheung et al., 2013; Robaina et al., 2017; Scheunemann et al., 2018), rice(Lakshmanan et al., 2013; Chatterjee and Kundu, 2015; Chatterjee et al., 2017), maize (Simons et al., 2014; Seaver et al., 2015; Bogart and Myers, 2016), tomato (Colombié et al., 2015; Yuan et al., 2016; Shameer et al., 2020), and potato (Botero et al., 2018). However, there is still no network model of tobacco, although it is one of the most important economic crops and a model for fundamental biology as well as an excellent chassis for plant synthetic biology. Tobacco has many advantages, such as high biomass, versatile metabolism (more than 4,000 producible chemical compounds), high protein expression (up to more than 40% of its biomass), mature agricultural and laboratory culture systems, as well as efficient techniques for rapid transient expression and chloroplast transformation (Bock, 2014; Azizi et al., 2015). Tobacco has been widely used for the heterologous synthesis of dozens of natural plant compounds, such as artemisinin, anthocyanins, etoposide aglycone, and taxadiene-5-ol (Farhi et al., 2011; Lau and Sattely, 2015; Appelhagen et al., 2018; Li et al., 2019; Zhu et al., 2021). In spite of its importance, to our best knowledge, no GSMN of tobacco has been published to date. Tobacco GSMN is desirable and will help to systematically understand and engineer tobacco metabolism. In this study, our first aim was to initially construct a high-quality GSMN for tobacco.

*In vitro* culture is a widely used technique for plant cultivation. It is an effective means for rapid clonal propagation of many valuable horticultural and pharmaceutical plants, while also offering a conventional and effective experimental system for plant physiology research. However, multiple specific effects of *in vitro* system on plant physiology could easily be overlooked or underestimated, such as the exogenous sugar supply, high humidity in compact and closed culture vessel, inefficient gas exchange, and sometimes low irradiance (Desjardins, 1995; Pospóšilová et al., 1999; PospÃ et al., 2007; Desjardins et al., 2009; Kozai, 2010; Yaseen et al., 2013). The compact space and high humidity will affect the water potential, transpiration rate, stomatal development and function, while sometimes also inducing stress and defense responses (Hsiao, 1973; Casanova et al., 2008; Ivanova and Van Staden, 2010). The poor ventilation can affect the carbon and oxygen balance, photosynthesis and respiration, as well as the accumulation of volatile secondary metabolites (Cournac et al., 1991; De Souza et al., 2008; De Oliveira et al., 2021). The low irradiance could affect light energy capture and utilization, the synthesis of photosynthetic pigments, photomorphogenesis, and biosynthesis of secondary metabolites (Kitaya et al., 1995; Kadleček et al., 2003; Eckstein et al., 2012). The exogenous sugar supply could affect the carbon, energy and osmotic balance, while also serve as the important signaling molecule to alter plant metabolism (Chanemougasoundharam et al., 2004; Choi et al., 2004; Badr et al., 2011; Xiao et al., 2011; Ševčíková et al., 2019). Therefore, the impact of *in vitro* cultivation on plant metabolism is a complex and multifaceted topic. Many factors and their interactions in *in-vitro* system could cause metabolic alterations or disturbances, and subsequently influence the overall metabolic profile of the plant. However, few studies embarked on a systemic and global elucidation of overall plant metabolic variations. Therefore, it is unclear what impact *in vitro* cultivation may have on overall plant metabolism. Genome-scale metabolic networks (GSMNs) are indispensable tools for the systematic characterization of metabolic behaviors. Metabolome and transcriptome analyses could globally elucidate overall metabolic changes and the underlying regulatory mechanisms. Therefore, combination of genome-scale metabolic networks and omics analysis is an excellent method for systematically elucidating the impact of *in vitro* cultivation on plant metabolism. In plants, omics data were successfully integrated with the *Arabidopsis* GSMN and rice GSMN to illustrate their metabolite acclimation profiles under different conditions (Töpfer et al., 2013; Töpfer et al., 2014; Lakshmanan et al., 2015). Similarly, we aimed to combine GSMN with omics data to uncover the metabolic heterogeneity across various *in vitro* culture conditions.

The aim of this research is to systematically elucidate the impact of *in vitro* cultivation on tobacco metabolism through GSMN-integrated multiomics analysis. Firstly, we initially constructed a genome-scale metabolic model of tobacco. Secondly, we detected the growth and photosynthetic differences between soil-grown tobacco and *in-vitro* tobacco. Thirdly, metabolome and transcriptome analyses were performed to identify the variations in metabolites and metabolic pathways between *in-vitro* and soil-grown tobacco plants. To systematically elucidate the impact of *in-vitro* cultivation on tobacco metabolic network, we combined model and omics data to elucidated metabolic network signatures of *in-vitro* tobacco plants, including photosynthesis, the Calvin and TCA cycle, as well as sucrose and N metabolism. This work allows us to systematically understand tobacco metabolism and the impact of *in vitro* cultivation, enabling us to efficiently utilize *in-vitro* techniques for plant cultivation and metabolic engineering.

## Results and discussion

### A genome-scale metabolic model of tobacco

**Figure 1:**
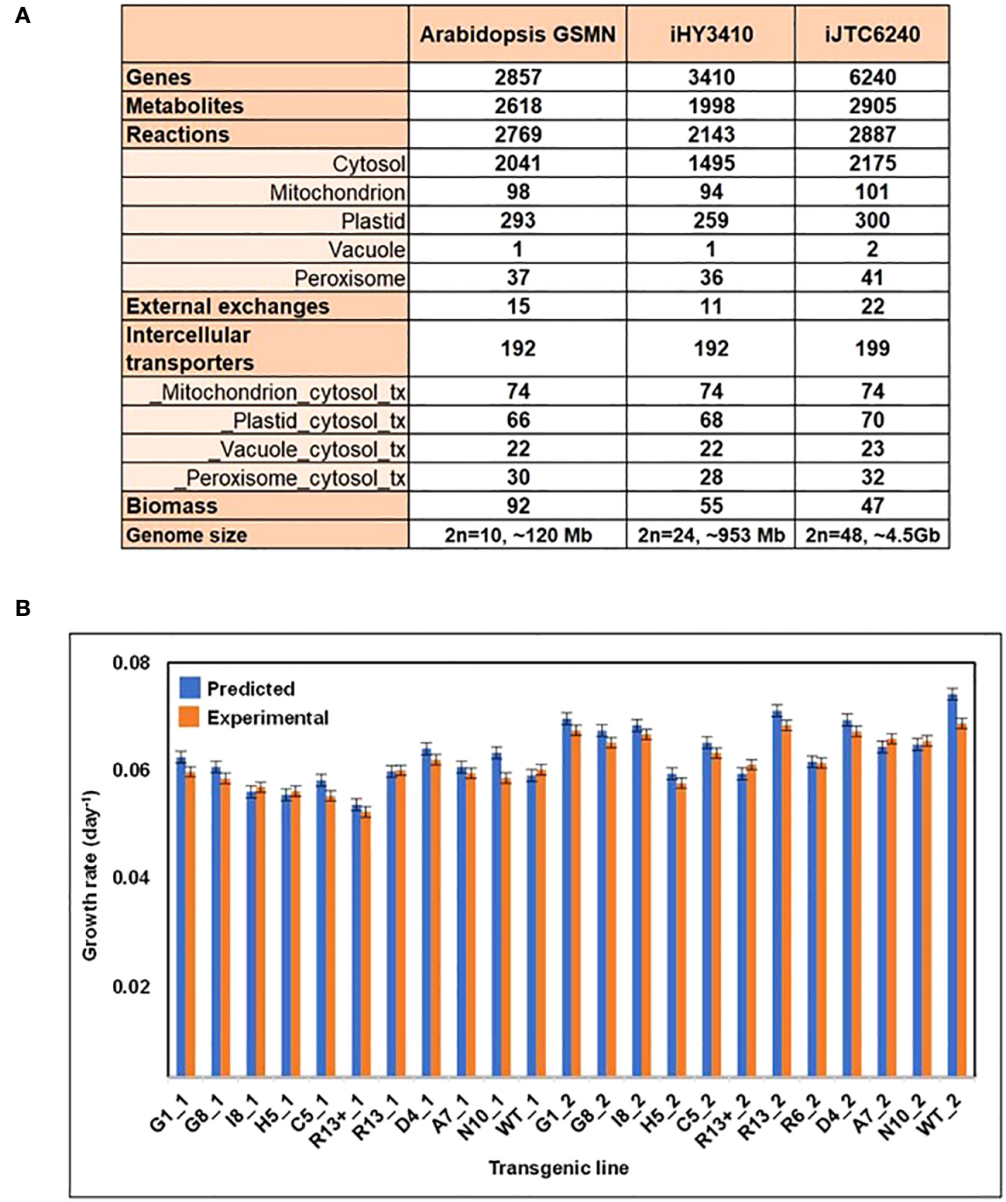
Overview and phenotype predictions of iJTC6240. **(A)** Comparison of model characteristics and reaction compartmentalization assignments between the *Arabidopsis* GSMN, iHY3410 and iJTC6240. **(B)** Comparison of tobacco transgenic line-specific phenotype predictions and experimentally observed growth rates. ‘_1’ and ‘_2’ as suffixes to the transgenic line names indicate that was obtained in 2012 and 2013 respectively. The experimental data was obtained from Jiang et al. (2018).

To systematically understand and engineer tobacco metabolism, we firstly had to reconstruct a genome-scale metabolic model (GSMN) for tobacco. To our best knowledge, this is the first reported tobacco model. The metabolic network was based on information from Ntabacum_Tn90Cyc (Plant Metabolic Network; https://plantcyc.org/content/ntabacumtn90cyc-3.0), Kyoto Encyclopedia of Genes and Genomes (KEGG), and other literature sources. Our tobacco GEM, named iJTC6240, accounts for 6,240 genes, 2,887 reactions, and 2,905 metabolites localized across five intracellular compartments, including cytosol, mitochondrion, plastid, vacuole, and peroxisome (**Figure 1A**). Among the 2,887 reactions, 2,619 are involved in biochemical conversions, while 199 are responsible for metabolite transport between the subcellular compartments, and there are 22 reactions that mediate the exchange with the environment. The inclusion of GPR associations in iJTC6240 allowed us to map the transcriptome data to contextualize the model for further analysis.

The model properties of iJTC6240 were compared with those of two plant GEMs, iHY3410 (Yuan et al., 2016) and *Arabidopsis* GEM (Cheung et al., 2013). **Figure 1A** summarizes the overall comparison of model characteristics and compartmental reactions between models. iJTC6240 collected more reactions, metabolites, and especially more genes than the other two models, which may be due to the larger genome of tobacco. More metabolic reactions are distributed in cytosol, mitochondria, plastid, vacuole and peroxisome compartments with various proportions. In addition to a single vacuole reaction found in both of iHY3410 and *Arabidopsis* GEM, also the same one, iJTC6240 collected one more vacuole reaction, whose subcellular location was validated in the literature. The reaction ‘RXN-10938_v’ describes the hydrolysis of phosphatidylinositol 3,5-bisphosphate (PtdIns(3,5)P2), which is essential for maintaining vacuolar morphology and function (Bonangelino et al., 2002). Consistently, the phosphoinositide phosphatase that catalyzes this reaction was reported to be localized to the vacuolar membrane (Rudge et al., 2004). A great number of metabolites and reactions unique to our tobacco model, that have no counterpart in *Arabidopsis* or tomato, including nornicotine and methylnicotinamide involved in nicotine metabolism. In tobacco, nicotine accounts for 95% of the total alkaloid content; tomatine, solanine and esculeoside, which were known as steroidal glycoalkaloids (SGAs) that are specialized metabolites produced by *Solanaceae* species.

After the GEM reconstruction, we performed several tests to validate its predictive capabilities. Firstly, the model was evaluated by simulating known metabolic functions, including absorption of PSI and PSII photons during photosynthesis, as well as the biosynthesis and degradation of secondary metabolites, such as alkaloids and terpenoids. Secondly, the ability of iJTC6240 to predict tobacco growth rates was tested. We utilized experimental photosynthetic capacity (Jiang et al., 2018) to constrain the model, and the model was validated by fairly correlation between the predicted and experimental growth rates (**Figure 1B**).

### *In-vitro* tobacco showed slower growth and less biomass than soil-grown plants

**Figure 2:**
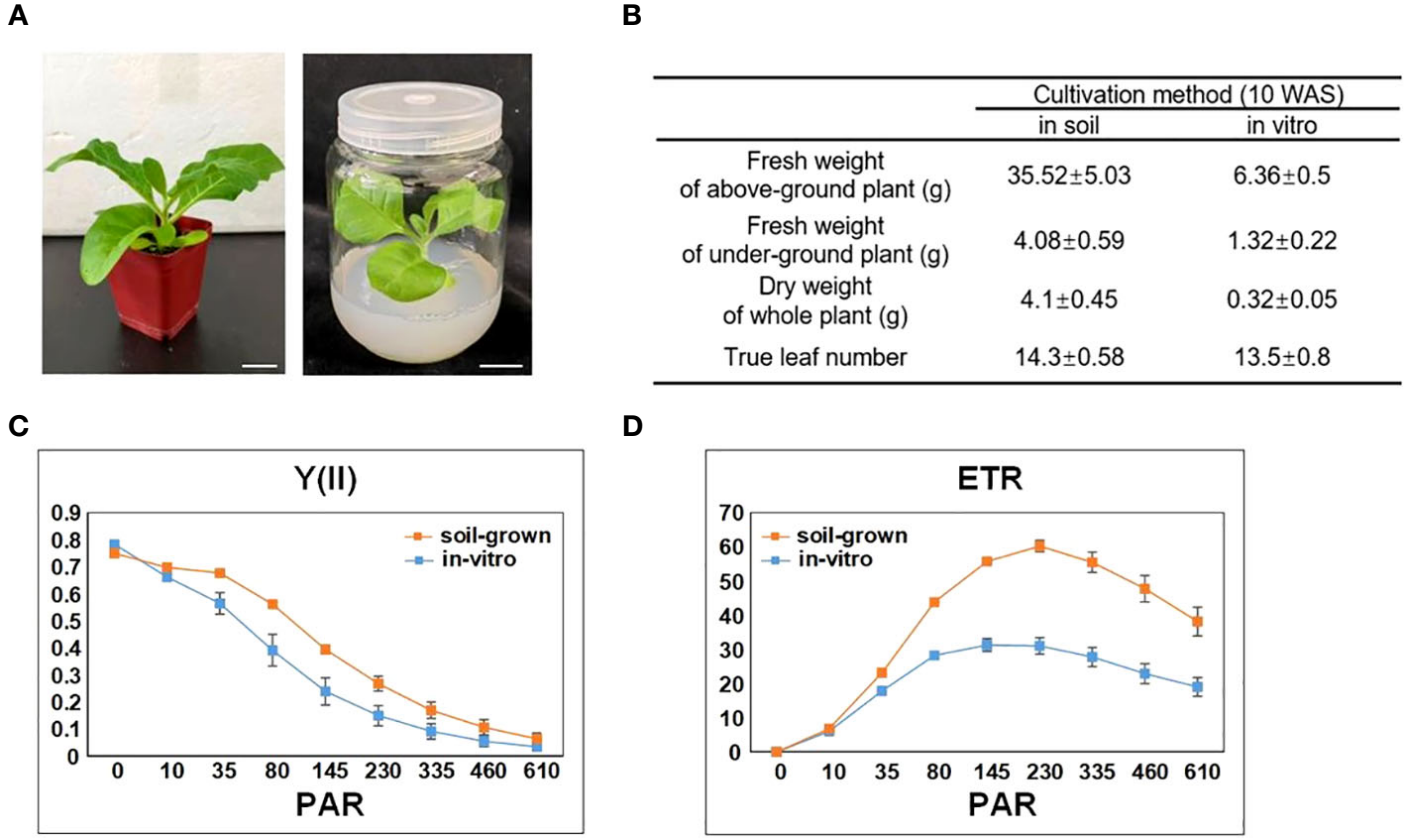
Impact of *in vitro* cultivation on tobacco growth and photosynthesis. **(A)** Tobacco plants cultivated in soil and *in vitro* (with 3% sucrose in the medium), both with 7 true leaves (approximately 5 WAS). Scale bars: 2 cm. **(B)** The growth difference between soil-grown and *in-vitro* cultivated tobacco plants. **(C)** The effective quantum yield of photochemical energy conversion in PSII (Y(II)). **(D)** The relative electron transport rate (ETR) of PSII. PAR denotes photosynthetically active radiation. Data are the means ± SE (n = 6). The orange color indicates the photosynthetic parameters of soil-grown leaves, while the blue color indicates the photosynthetic parameters of *in-vitro* leaves.

To reflect the general impact of *in-vitro* cultivations on tobacco, we selected the most common conditions to cultivate tobacco plants *in vitro* and in soil (as described in the methods section). The seeds germinated approximately 1 week after sowing (WAS), and the germinated seedlings were transplanted into soil or *in vitro*. The *in-vitro* plants grew much slower than that in soil, but the true leaf number of both tobacco plants was almost the same during all stages of development (**Figures 2A, B**). We compared a number of growth parameters between *in-vitro* and soil-grown tobacco plants at 10 WAS, at which time the top of the *in-vitro* plants reached the cap of the culture vessel. *In-vitro* tobacco was much smaller and its fresh weight was approximately one fifth of the soil-grown fresh weight. Moreover, the *in-vitro* dry weight was only one twelfth of the soil-grown dry weight, indicating that the water content of the *in-vitro* plants was much higher than that of soil-grown plants. The numbers of true leaves were similar between *in-vitro* and soil-grown tobacco plants at 10 WAS, and none of the plants developed flower buds during the study period (**Figure 2B**).

### Suppressed photosynthesis in *in-vitro* tobacco plants

We measured the leaf chlorophyll fluorescence parameters to explore the photosynthetic system of *in-vitro* and soil-grown plants. The Fv/Fm, ratio which represents maximum quantum yield of PS II photochemistry, also indicates the stress level of the plants. According to this measure, the *in-vitro* and soil-grown plants had a similar photosynthetic capacity (0.78 ± 0.01 in soil-grown vs. 0.75 ± 0.005 in *in-vitro* plants; n=6). To further assess the differences in the photosynthetic efficiency, the effective quantum yield of photochemical energy conversion in PS II (Y(II)) and relative electron transport rate (ETR) were calculated for different light intensities (PAR). **Figure 2C** showed the Y (II) curve was generally lower in *in-vitro* tobacco plants than that in in-soi ones, and gradually decreased to nearly 0 µmol at 610 µmol photons m^-2^s^-1^ following light intensity increased. The ETR curve under *in-vitro* condition was also lower than that under soil-grown condition (**Figure 2D**), and it gradually increased from 0 to nearly 60.22 µmol in soil-grown and 31.04 µmol in *in-vitro* at 230 µmol photons m^-2^s^-1^ (PAR), once reached this level, ETR decreased. Collectively, these results indicated the suppressed photosynthetic capacity under *in-vitro* condition.

### Metabolic variations between *in-vitro* and soil-grown tobacco plants revealed by metabolome, transcriptome and amino acid data

The above results showed that *in-vitro* cultivation inhibited the growth of tobacco compared to soil-grown cultivation. To study the impact of *in-vitro* cultivation on tobacco metabolism and globally detect the metabolic variations between *in-vitro* and soil-grown tobacco plants, we performed metabolome and transcriptome analyses for 10-WAS *in-vitro* and soil-grown tobacco plants.

We generated transcriptome sequencing data for *in-vitro* tobacco and soil-grown tobacco plants, each with three biological replicates. After filtering out low-quality reads from the raw data, a total of 37.77 GB clean data was generated, and 5.64−6.74 GB for each sample was obtained. The GC content was ranging from 43.20% to 43.50%, and the percentages of Q30 bases were over 92.75%. The mapped efficiency of different samples with reference genome ranged from 94.64% to 95.12% (**Table S1**). The assembly resulted in 88,878 unigenes, of which 13,890 differentially expressed genes (DEGs) were identified between *in-vitro* and soil-grown plants (8,519 downregulated, 5,371 upregulated) were identified from transcriptomic data, and their GO functional annotation is shown in **Supplementary Figure S1**.

To detect the difference in metabolite abundance and type between *in-vitro* and soil-grown tobacco plants, a widely targeted metabolomics analysis was conducted in triplicates. From the overlapping map of total ion current (TIC) of the quality control (QC) sample, it could be observed that there was a high overlap in the curve of the total ion flow, and the retention time and peak intensity remained consistent. This suggested that the signal stability for the same sample was good when the mass spectrometry was detected at different times (**Supplementary Figure S2**). In this study, a total of 680 metabolites were identified across all samples. All metabolites can be categorized into 12 classes, including lipids (131), phenolic acids (91), flavonoids (89), alkaloids (77), amino acids and derivatives (69), organic acids (47), nucleotides and derivatives (40), lignans and coumarins (25), terpenoids (22), tannins (5), quinones (3) and other metabolites (81). UPLC-ESI-MS/MS-based metabolomic and OPLS-DA (orthogonal partial least squares-discriminant) analyses (**Supplementary Figure S3**) identified 390 differentially accumulated metabolites (DAMs), 207 downregulated and 183 upregulated.

### Cultivation-condition-specific metabolites

Among the 183 upregulated DAMs, 25.7% exhibited abundance in *in-vitro* tobacco plants but were not detected in soil-grown ones. Some of these *in-vitro*-specific metabolites were environmental stress markers or stress response compounds, such as malate and tomatidine (**Table S2**). The dicarboxylic acid malate has a multitude of functions in plant physiology and metabolism, and it was characterized as a major constituents of soluble carbon fraction involved in osmotic adjustment (Finkemeier and Sweetlove, 2009). The high accumulation of malate may help to cope with the ambient osmotic potential of *in-vitro* culture media. It is reported that the increased levels of tomatidine resembled toxic phenotypes, such as growth retardation (Itkin et al., 2011).

Among 207 downregulated DAMs, 16.4% exhibited abundance only in soil-grown tobacco plants, some of which may reflect the specific culture condition and metabolic responses, such as capsianosides and planteose. Capsianosides were pinpointed as metabolites related to insect resistance (Macel et al., 2019), reminiscent of the flying insects in the climate chamber. Planteose is regarded as a temporary sugar storage compound in plants (Klages et al., 2004). The condition-specific accumulation of planteose may indicate that soil-grown tobacco plants could fix excess carbohydrates through photosynthesis, while *in-vitro* tobacco plants could not, implying a suppression of photosynthesis in the *in-vitro* tobacco plants.

### Cultivation-condition-specific enriched metabolic pathways and metabolite classes

**Figure 3:**
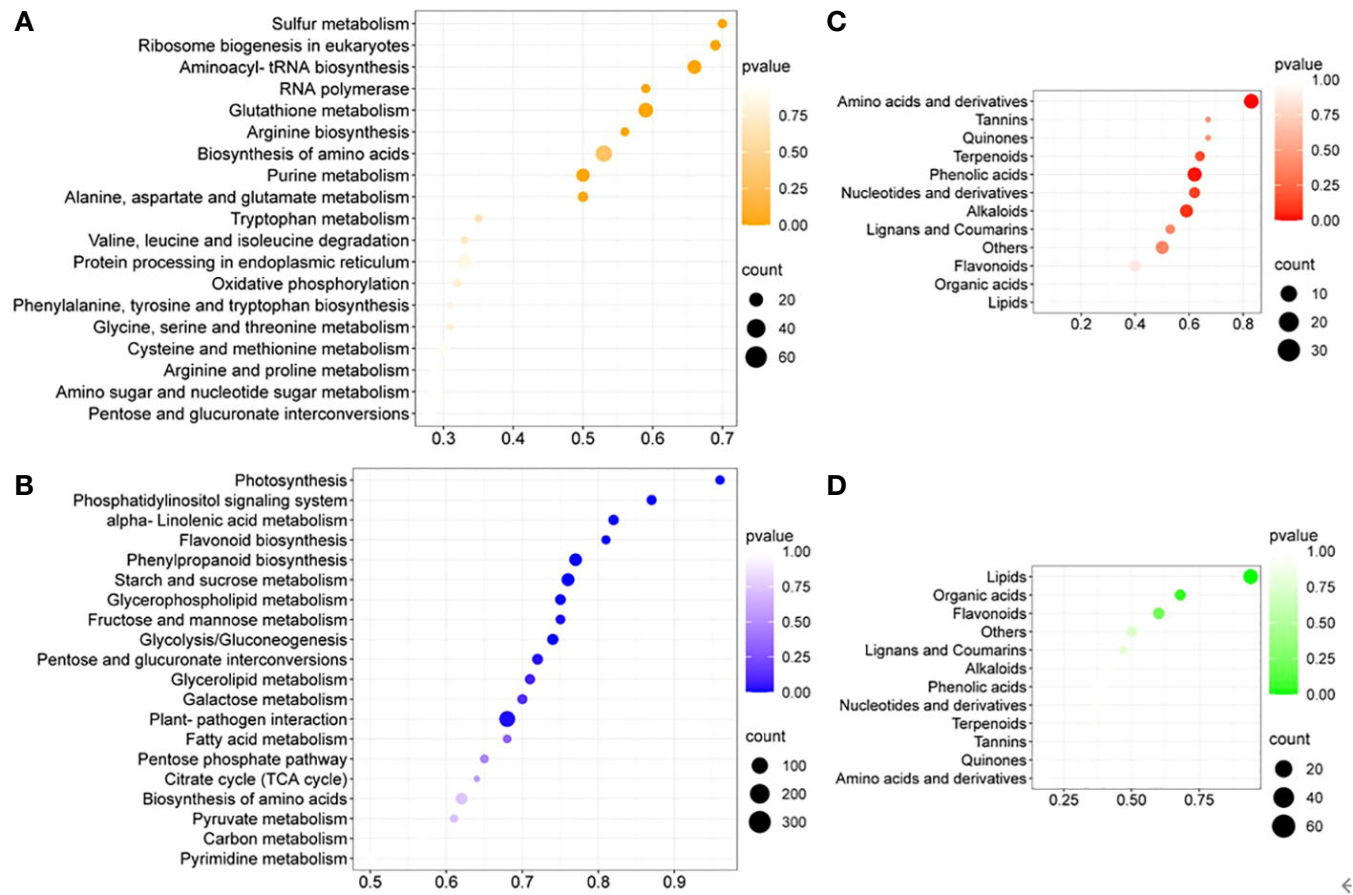
The enrichment analysis of DEGs and DAMs between different culture conditions. **(A)** The top twenty KEGG pathways enriched among upregulated DEGs, **(B)** the top twenty KEGG pathways enriched among downregulated DEGs; **(C)** The enrichment analysis of UpSet DAMs, **(D)** the enrichment analysis of DownSet DAMs.

To further identify cultivation-condition-specific metabolic pathways, KEGG pathway enrichment analysis of DEGs was performed (**Figure 3**). Many amino acid metabolism related pathways were upregulated, supported by enriched upregulated DEGs related to aminoacyl-tRNA synthesis and amino acids biosynthesis (**Figure 3A**). The metabolic pathways of alpha-linolenic acid, glycerophospholipid, glycerolipid and fatty acid, which are involved in lipid metabolism, were enriched among the downregulated DEGs (**Figure 3B**). The primary carbon metabolism was generally downregulated in *in-vitro* tobacco plants compared to soil-grown ones, since the photosynthesis, glycolysis/gluconeogenesis, pentose phosphate pathway (PPP) and TCA cycle, as well as its closely related pathways, such as the metabolism of starch, sucrose, fructose, mannose, pentose, galactose and pyruvate were all downregulated. At the same time, many downregulated plant secondary metabolic pathways were enriched under *in-vitro* condition, including the phosphatidylinositol signaling system, flavonoid biosynthesis, and phenylpropanoid biosynthesis. Collectively, enrichment analysis of DEGs suggested that in *in-vitro* tobacco plants amino acid metabolism was up regulated, while lipid metabolism was attenuated, and the growth inhibition was likely due to the lower activity of primary carbon metabolism and related pathways. In addition, the secondary metabolism of *in-vitro* tobacco plants was also weakened compared to soil-grown ones.

DAMs were utilized for the enrichment analysis based on the metabolic classification. The upregulated amino acids and derivatives were enriched among the DAMs with the greatest enrichment degree (**Figure 3C**). In addition, lipids were enriched among the DownSet of DAMs with the greatest enrichment degree (**Figure 3D**).

### The content of amino acids increases significantly under *in-vitro* tobacco plants

Compared to soil-grown tobacco, a majority of primary metabolic pathways were downregulated in *in-vitro* tobacco plants, but the synthesis and accumulation of amino acids was upregulated indicted by analysis of metabolome and transcriptome, which were relative quantification analyses. A total of 5 amino acids and derivatives exhibited significant abundance in *in-vitro* plants but were not detected in soil-grown tobacco, such as homocysteine. Homocysteine is a non-proteinogenic amino acid and is considered to response to environmental stress (Watanabe et al., 2021). Only one metabolite in the term of amino acid and derivatives, L-theanine, exhibited significant abundance in soil-grown tobacco while it was not detected in *in-vitro* plants. It was reported that theanine content significantly increased when attacked by *Ectropis grisescens* (Liao et al., 2019), indicating that the soil-grown plants may have been exposed to insects. Relative quantification results based on metabolome showed the overall levels of the 20 common proteinogenic amino acids were higher in *in-vitro* tobacco plants (**Table S2**).

**Figure 4:**
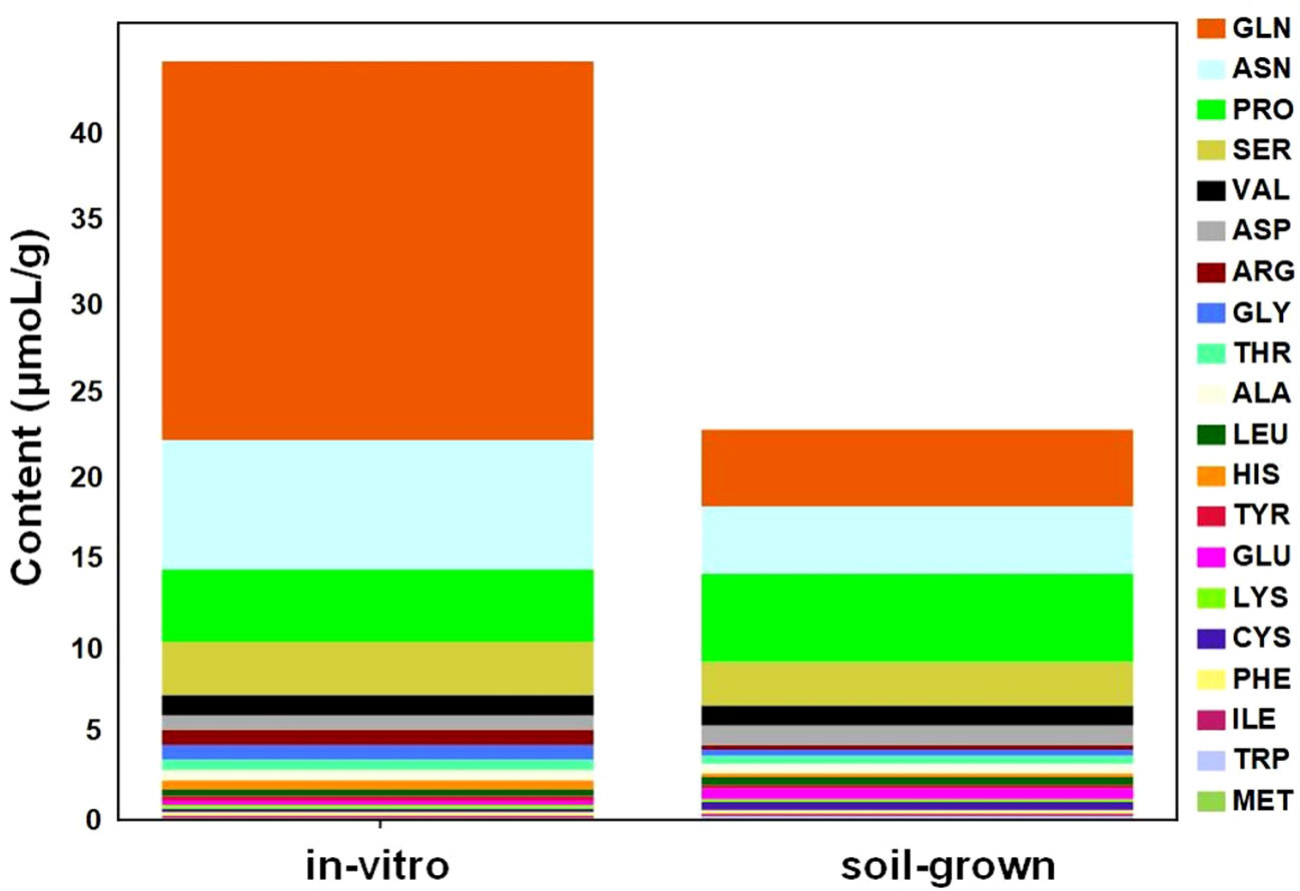
Changes in the absolute concentrations of common amino acids between *in-vitro* and soil-grown conditions.

To further quantitatively compare the common amino acids in plants from different culture conditions, automatic amino acid analyzer was utilized to determine the absolute concentrations of free common amino acids. The overall level of free amino acids under *in-vitro* condition was higher than soil-grown condition, which revealed approximately 2-fold higher amino acid levels in the *in-vitro* plants (**Figure 4**). The top 5 most highly accumulated free amino acids, accounting for over 80% of the total content of common amino acids, were distributed in different metabolic branches. For example, Gln and Pro are derived from α-ketoglutarate, Asn is derived from oxaloacetate, Ser is derived from 3-phospho-glycerate and Val is derived from pyruvate. Additionally, four of the top 5 most highly accumulated amino acids were more abundant in *in-vitro* than in soil-grown plants, including Gln (4.92-fold), Asn (1.92-fold), Ser (1.21-fold), and Val (1.19-fold), but not Pro (0.82-fold). Among these, Ser, Val and Pro were also detected in the metabolomic data, and their metabolic peak areas were consistent with the measured absolute concentrations. Notably, the concentration of Gln, which was not detected in the metabolomic data, was much higher than that of the other amino acids and was primarily responsible for the significant upregulation of amino acids in *in-vitro* tobacco plants.

In order to further exclude the influence of developmental status (such as flowering transition) on plant metabolism, 7-WAS tobacco metabolic variations between two cultivations were also tested except for 10-WAS tobacco. We measured the concentrations of amino acid, one of the most characteristic metabolic variations, for 7-WAS tobacco (**Supplementary Figure S4**), and amino acid concentrations increased significantly under *in-vitro* condition, the pattern was consistent with the results of 10-WAS plants. These results indicated the metabolic variations we detected were mainly due to the different cultivation systems.

### Metabolic network signatures of *in-vitro* tobacco plants unraveled by omics-based modeling

**Figure 5:**
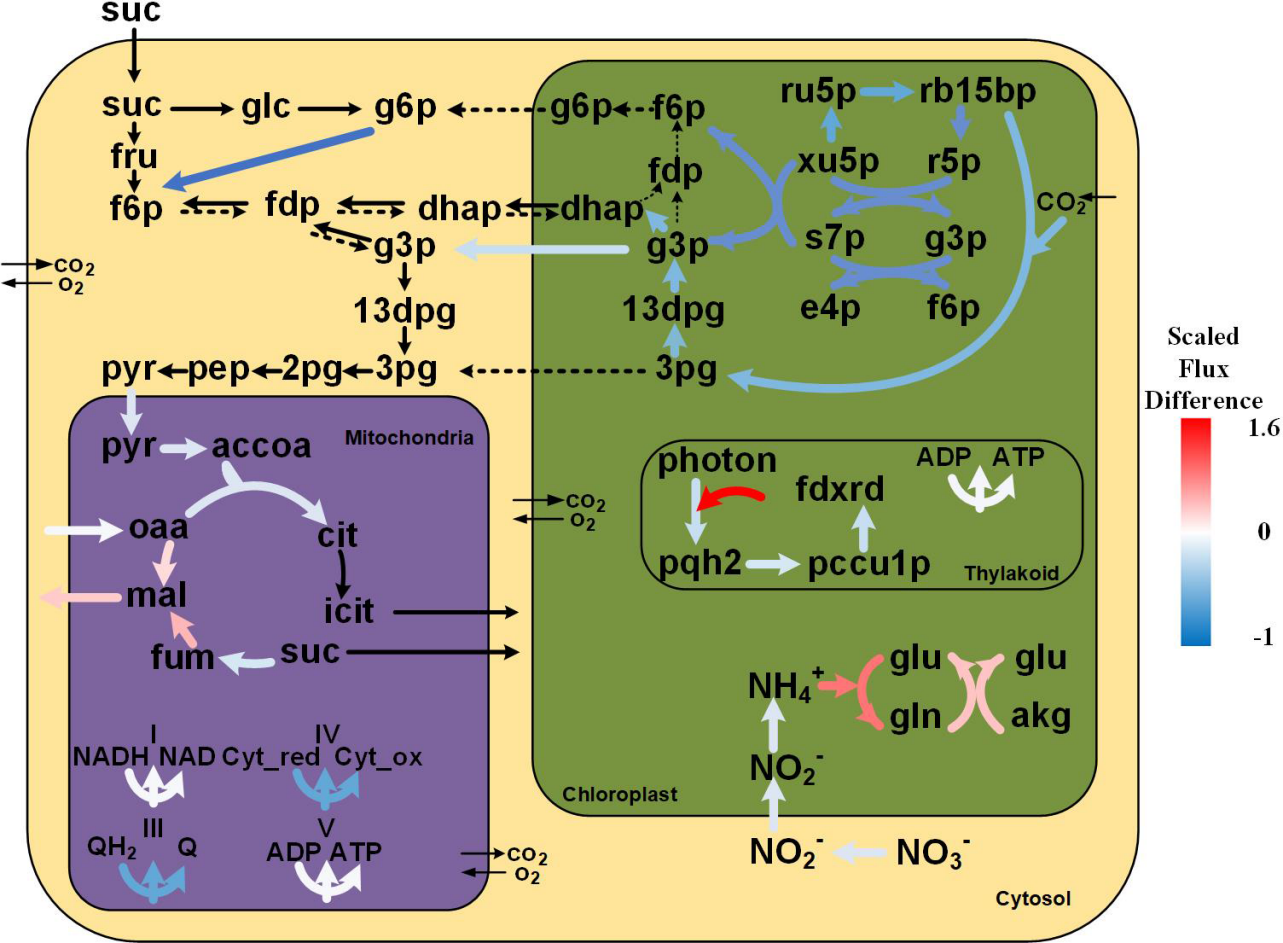
Flux distribution of the primary metabolism of tobacco under soil-grown and *in-vitro* conditions. The black bold arrows indicate reactions carrying non-zero fluxes in *in-vitro* plants while having zero fluxes in soil-grown plants; the black dotted arrows indicate reactions carrying non-zero fluxes in soil-grown plants while having zero fluxes *in-vitro* based on FBA solutions. The differences in the flux values under the two conditions were calculated using the log10 scale fold changes of *in-vitro*/soil-grown values as a reference. Metabolite abbreviations are as follows: suc, sucrose; glc, glucose; g6p, glucose 6-phosphate; fru, fructose; f6p, fructose 6-phosphate; fdp, fructose 1,6-bisphosphate; dhap, dihydroxyacetone phosphate; g3p, glyceraldehyde 3-phosphate; 13dpg, 3-phospho-glyceroyl phosphate; 3pg, 3-phospho-glycerate; 2pg, glycerate 2-phosphate; pep, phosphoenolpyruvate; pyr, pyruvate; accoa, acetyl-coa; cit, citrate; icit, isocitrate; fum, fumarate; mal, malate; oaa, oxaloacetate; Cyt_red, Cytochromes c_reduced; Cyt_ox, Cytochromes c_oxidized; QH2, ubiquinol; Q, ubiquinone; ru5p, ribulose 5-phosphate; xu5p, xylulose 5-phosphate; s7p, sedoheptulose 7-phosphate; e4p, erythrose 4-phosphate; rb15bp, ribulose 1,5-bisphosphate; r5p, ribose 5-phosphate; pqh2, plastoquinol; pccu1p, plastocyanin reduced; fdxrd, ferredoxins reduced; gln, glutamine; akg, 2-oxoglutarate; glu, glutamate.

To further explore how different culture conditions influence growth, we examined the internal flux distributions under soil-grown and *in-vitro* conditions. The transcriptome data and measured amino acid concentrations under *in-vitro* and soil-grown conditions were used to calculate constraints to GEM, leading to context specific models. The flux balance analysis results showed that the growth rate of the tobacco plants was lower under *in-vitro* than under soil-grown. Notably, multiple crucial metabolic pathways were downregulated under *in-vitro* condition, including photosynthesis, the Calvin cycle, glycolysis, and tricarboxylic acid cycle (**Figure 5**). The slower growth and less biomass under *in-vitro* condition may be related to the overall downregulation of primary carbon metabolism. By contrast, the cyclic electron flow (CEF) and the glutamine synthetase (GS)/glutamine-2-oxoglutarate aminotransferase (GOGAT, also known as glutamate synthase) cycle were upregulated under *in-vitro* condition, and the metabolomic data also showed that the concentration of Gln was higher than that under soil-grown. After normalized by the biomass rate, more ATP was produced by proton pumps (1.5-fold), while less NADH/NADPH (by 12% and 0.18%, respectively) was produced under *in-vitro* condition.

### Photosynthesis

Photosynthetic organisms use various electron flow pathways to generate redox power from light energy in the form of NADPH. The linear electron flow (LEF) pathway, where the electrons flow from PSII to PSI through Cytochrome b6f complex and ferredoxin NADP^+^-oxidoreductase, is the most extensively used pathway (Allen, 2002). Additionally, plants also possess other alternative electron flow (AEF) pathways, such as CEF, which provides acclimatory plasticity for the photosynthetic machinery. In the CEF process, electrons are recycled to generate a proton gradient that drives the synthesis of ATP, which is essential for balancing the energy demand. Although the physiological roles of CEF pathways are not clear, they are suggested to function alongside LEF (Allen, 2003).

The simulations revealed that both the LEF and CEF pathways carried noticeable fluxes under soil-grown and *in-vitro* conditions, suggesting that the CEF pathway is possibly always operational. Interestingly, the predicted rate of photosynthesis under *in-vitro* condition was much more dependent on the CEF pathway than under soil-grown condition. In fact, the corresponding energy generated by the CEF pathway under *in-vitro* condition was 42.9-fold higher than in soil-grown plants. The CEF pathway under *in-vitro* condition, rather than LEF, was used increasingly to generate more energy, that may response to the lower reducing power demand in the Calvin cycle because of low CO_2_ uptake, as well as the need to scavenge excess reducing power, which is cytotoxic. Additionally, considerable ATP was needed in a multitude of metabolic pathways, including Gln biosynthesis by glutamine synthetase and sucrose import via transmembrane transporters. Generally speaking, the CEF pathway carried the higher fluxes, highlighting its flexibility and also suggesting that the CEF pathway may contribute to the adaptation of energy demand under *in-vitro* condition.

### Calvin cycle

The Calvin cycle exhibited appreciable lower fluxes (by 43%) in *in-vitro* plants compared to soil-grown plants, which was attibuted to the lower CO_2_ uptake. In both simulations, the Calvin cycle was driven by the supply of CO_2_ from cytosol to plastid and was subsequently withdrawn in the form of 3-phosphoglycerate (3PG), that can be transported to cytosol. The 3PG in the cytosol was utilized for energy production through cytosolic glycolysis and the TCA cycle, in agreement with previous reports (Lakshmanan et al., 2013). In this regard, the flux analysis results indicated reduced fluxes (by 76%) under *in-vitro* condition, whereby the 3PG in the plastid was utilized to produce 1,3-bisphosphoglycerate (13DPG) instead of energy generation, with a decreased flux (by 45%). 13DPG was converted into glyceraldehyde 3-phosphate (G3P) that participated in PPP and gluconeogenesis. Under *in-vitro* condition, the plastidic dihydroxyacetone phosphate (DHAP) produced from G3P by fructose-bisphosphate aldolase was transported to cytosol to synthesize Fru-1,6-bisP, while under soil-grown condition it was utilized to synthesize plastidic G6P, which was transported to cytosol to enter Embden-Meyerhof-Parnas (EMP) pathway.

### TCA cycle and oxidative phosphorylation

The model simulation revealed an incomplete TCA cycle under both conditions. As there are multiple cytosolic isoforms of the enzymes that catalyze the mitochondrial TCA reactions, it stands to reason that some mitochondrial isoforms were not used in our model prediction (Sweetlove et al., 2010), as for example succinyl-CoA synthetase operated in the form of a cytosolic bypass in the prediction. The operation of an incomplete TCA cycle in the light by our tobacco model is supported by a study in French bean leaves (*Phaseolus vulgaris*), which indicated that the TCA cycle was almost completely inhibited under light (Tcherkez et al., 2005). Similarly, Poolman et al. (2013) reported that the TCA cycle did not function as a conventional cycle in rice under high light (Poolman et al., 2013).

In the simulation of soil-grown tobacco, no flux was observed from citrate to succinate, while under the *in-vitro* condition, carbon flux enters the cycle at two different points: (1) acetyl-CoA from pyruvate and (2) succinate from cytosol. The TCA cycle of *in-vitro* tobacco plants was impaired (flux reduction by 77%), with an increase of malate production from fumarate (2.9-fold) and from oxaloacetate (by 68%). The malate highly produced by TCA cycle in mitochondria was then transported to plastids, where it was converted into oxaloacetate by malate dehydrogenase (NAD) to provide reducing power for the GS/GOGAT cycle, while the oxaloacetate was utilized to form Asp.

The fluxes within mitochondrial respiration were relative mild reduced under *in-vitro* condition (by 92%) with a 90% flux of oxidative NADH as the first step of the respiratory chain. Additionally, succinate dehydrogenase (SDH, also known as Complex II) has a dual function as a component of the TCA cycle and the electron transport chain (ETC). The flux through SDH was comparable with its TCA cycle flux (76.7%).

### Sucrose metabolism

Since sucrose (Suc) was imported from the surrounding medium under *in-vitro* condition, noticeable levels of ATP were consumed for this active transmembrane transport process. The sucrose invertase (INV) was predicted to degrade Suc into fructose and glucose, while that is not an energetically efficient way to break down Suc when compared to the cleavage of Suc by sucrose synthase (SUS) (Magneschi and Perata, 2009). Subsequently, fructose and glucose are converted into G6P, which flows into the EMP pathway, while under soil-grown condition, G6P that drives EMP is supplied by its plastidic isoform and is derived from F6P, which synthesizes via the pentose phosphate pathway and the hydrolysis of fructose-1,6-bisphosphate.

### N metabolism

In our model, we considered that tobacco can take up NH_4_^+^ and/or NO_3_^-^ as nitrogen sources. After uptake, nitrate is firstly reduced into nitrite by nitrate reductase (NR) in cytoplasm, after which nitrite is transported into plastid and reduced to ammonium by plastidic nitrite reductase (NiR). In this regard, the flux analysis results indicated that this route exhibited reduced fluxes (by 75%) under *in-vitro* condition, and the resulting lower consumption of reducing power may be related to its lower supply from photosynthetic light reactions, and a greater proportion were shifted toward energy generation.

Interestingly, ammonium assimilation, by which inorganic N is converted into organic N, was higher under *in-vitro* condition. The ammonium assimilation in plastid is accomplished by the GS/GOGAT cycle, where GS catalyzes glutamine (Gln) synthesis by incorporating a molecule of ammonium into glutamate (Glu) in an ATP-dependent manner, while GOGAT produces two molecules of Glu by transferring the amide from Gln to 2-oxoglutarate (2-OG). Plants possess two isoforms of GOGAT that differ in their electron donor for catalysis, NADH-GOGAT (EC 1.4.1.1) and ferredoxin (Fd)-GOGAT (EC 1.4.7.1), both of which are located in the plastids (Lea and Blackwell, 1993). Our model predicted that tobacco plants utilize the NADH-GOGAT to generate Glu. The resulting constraint-based flux analysis revealed that the flux through NADH-GOGAT (EC 1.4.1.1) was higher under in-vitro condition than that in soil-grown plants (2.14-fold). In higher plants, there are two types of GS with distinct subcellular localization, cytosolic GS1 and plastidic GS2. Our model predicted that glutamine was only produced by plastidic GS2 in both cases, and the flux was higher *in-vitro* than in soil-grown plants (4.91-fold). Collectively, it can therefore be seen that the higher activity of the GS/GOGAT cycle under *in-vitro* condition results in increased Gln synthesis at the expense of Glu, since the flux through GS was higher than through GOGAT. These findings may offer a reasonable explanation for the higher Gln but lower Glu content under *in-vitro* condition compared to soil-grown plants.

The composition of the culture medium is crucial for *in-vitro* cultivation. MS medium was originally designed for tobacco callus and is widely used for many plant species nowadays. MS medium contains macronutrients that include high levels of nitrate and organic additives, such as sugar, and vitamins. Sugar provides metabolic energy and carbon skeletons for the biosynthesis of the organic compounds required for cell growth and also act as an osmotic component in the culture medium. Stancato and Tucci (2010) emphasized that the accumulation of dry matter *in-vitro* plants is positively correlated with the sucrose content in the culture medium. However, high sucrose concentration added to the culture medium can cause starch and sucrose accumulation in the leaves, which can reduce the photosynthetic activity (Schmildt et al., 2015). In our study, the photosynthetic activity of *in-vitro* tobacco was suppressed, which may be attributed to an abundance supplement of sucrose [3% (w/v)] in the culture medium. The increasing nitrogen supplement could lead to an increase in the total nitrogen content in tissues. Sarimento et al. (1992) revealed that the application of increasing quantities of nitrogen enhances the accumulation of the free amino acids in grapevine explants. In this study, the content of some amino acids also increased significantly in *in-vitro* tobacco. Furthermore, in silico simulations showed *in-vitro* tobacco had a higher activity of the GS/GOGAT cycle fixing the substantial amounts of ammonium at the expense of Glu to produce Gln, which may be attributed to an abundance supplement of ammonium salts in the culture medium.

Compared to soil-grown tobacco, although there was additional carbon (sucrose) uptake, we noticed diminished carbon fixation in the Calvin cycle in our *in silico* investigation, that resulted in lower growth rate and less biomass under *in-vitro* tobacco. The Calvin cycle was subjected to energy and reducing power. Although ATP production was abundant, the generation of reducing power (NADH/NADPH) is markedly limited indicated by our systematical combined omics-based analysis. Here we gave three suggestions on how to improve the situation under *in-vitro* condition. Firstly, our model prediction showed a surprisingly high level of cyclic photophosphorylation, resulting in the reduction power being utilized for the generation of abundance energy. We proposed suppressing cyclic photophosphorylation and boosting non-cyclic photophosphorylation activity for this purpose. Additionally, our model survey also predicted increased flux from fumarate to malate but decreased subsequently in the TCA cycle. To improve the flux towards oxaloacetate and consequently the overall TCA cycle, we suggested that supplement the culture medium with appropriate pyruvate or organic acid (such as acetate, citrate) salts, which could potentially elevate the concentration of TCA metabolites intermediates and thereby drive the TCA cycle to generate reducing power. Finally, addition of glycerin into medium was supposed to help to alleviate the deficiency in reductive power.

To improve the suppressed photosynthesis in *in-vitro* tobacco than soil-grown tobacco, we proposed: Firstly, since we provided sufficient detail in iJTC6240 to define all individual electron transport reactions including photosystem II (PSII), photosystem I (PSI), and the cyclic/non-cyclic electron transfer, both photons utilized in PSII and PSI exhibited lower level of flux (by 74% and 67%, respectively) under *in-vitro* compared to soil-grown tobacco in our observations. We speculated that maybe an appropriately elevation of effective photons utilization that not inducing photoinhibition would confer improvements. Secondly, light quality serves as a pivotal factor affects various morphological processes in plants, it was reported that in blue light, photosynthesis was up-regulated (Lakshmanan et al., 2015). In that way, it was hypothesized that varying light wavelengths in chamber may modulate the suppressed photosynthetic processes *in-vitro*.

## Materials and methods

### Plant material, growth conditions and biomass determination

Tobacco (*Nicotiana tabacum* cv. Petit Havana) seeds were sterilized with 75% ethanol for 1 min, and then sterilized with 10% sodium hypochlorite for 20 min, rinsed with sterile water three times, and finally germinated on Murashige and Skoog (MS) agar plates containing 3% (w/v) sucrose in a climate chamber at temperature of 25°C with a light/dark cycle of 16 h/8 h and photon fluence of 186 μmol m ^−2^ s ^−1^. The germinated seedling was transplanted into pots containing soil/vermiculite mixture (1:1) or into 500 mL glass culture vessels (covered with gas-permeable caps) containing solidified MS medium (100 mL per vessel) with 3% sucrose. The fresh weight of the tobacco aboveground part was measured using an electronic balance. The dry weight was recorded after drying the samples in an oven at 65°C to constant weight.

### Nucleic acids extraction

Genomic DNA was isolated using a Super Plant Genomic DNA Kit (Tiangen Biotech, Beijing). Total RNA was extracted from 10-WAS plants using an RNAprep Pure Plant Plus Kit (Tiangen Biotech, Beijing). DNA and RNA degradation and contamination were monitored on a 1% (w/v) agarose gel. RNA purity and integrity were assessed using the NanoPhotometer^®^ spectrophotometer (IMPLEN, CA, USA) and the RNA Nano 6000 Assay Kit of the Agilent Bioanalyzer 2100 system (Agilent Technologies, CA, USA), respectively. RNA concentration was measured using the Qubit^®^ RNA Assay Kit in the Qubit^®^2.0 Flurometer (Life Technologies, CA, USA).

### Transcriptome analysis

The aboveground parts of three independent plants were collected and pooled as one transcriptomic sample, each sample with three biological replicates. Transcriptome analyses were performed for *in-vitro* and soil-grown tobacco plants. Qualified RNA from each samples were shipped on dry ice to the Biomarker Biotechnology Corporation (Beijing, China) for cDNA library construction and RNA sequencing. The library was prepared using the VAHTS^®^ Universal V8 RNA-seq Library Prep Kit for Illumina (Vazyme Biotech, Nanjing, China). Subsequently, the cDNA libraries were paired-end sequenced on an Illumina NovaSeq 6000 platform (Illumina, San Diego, CA, USA). Clean reads were obtained by removing reads containing adapters or ploy-N and low-quality reads from the original data. At the same time, the percentage of Q30 bases and GC-content of the clean data were calculated. All downstream analyses were based on clean data of high quality. After performing quality control, we obtained clean reads which were then mapped to the tobacco reference genome Ntab_TN90 (Sierro et al., 2014) using HISAT2 (Kim et al., 2015b). The mapped reads were subsequently assembled using StringTie (Pertea et al., 2015). The assembled genes were aligned against several databases, including Nr, Swiss-Prot, KEGG, COG, GO, KOG, eggNOG and Pfam, with BLASTX at an e-value cutoff of 1e–5 (Altschul et al., 1997). Gene expression levels were calculated based on the read counts and normalized by the fragments per kilobase of transcript per million fragments mapped (FPKM) method (Florea et al., 2013). DEGs were identified using the DESeq2 package (Love et al., 2014) using a false discovery rate (FDR)<0.05 and fold-change (FC) ≥2 or FC ≤ 0.5 as the filtering criteria.

### Metabolome analysis

Metabolite profiling was carried out using a widely targeted metabolomic method by Metware Biotechnology (Wuhan, China) for the same samples as transcriptome. Briefly, the freeze-dried plant samples were crushed using a mixer mill (MM 400, Retsch) and extracted with 70% methanol extraction solution. After centrifugation, the supernatant was filtrated and then analyzed using a UPLC-ESI-MS/MS system in conjunction with UPLC (Shim-pack UFLC Shimadzu CBM30A system) and MS (Applied Biosystems 4500 Q TRAP). The analytical parameters were set as the same as Shi et al., 2021. For metabolomics data, the primary results were identified by comparing the fragmentation patterns, accurate *m/z* value and retention time to the standards in the self-compiled database (MetWare, Wuhan, China) and the public databases (Metlin and Massbank). The characteristic ions of each metabolite were obtained by triple four-stage rod screening, the signal intensity of the characteristic ions was obtained in the detector, and MultiQuant software was used to integrate and correct the chromatographic peaks. All integration data for the chromatographic peak areas were exported and saved. To detect the repeatability of the samples, QC samples (mixed sample extracts) were inserted in every 10 test samples during the analysis. The accuracy and reproducibility of metabolite detection could be determined by using the overlapping display analysis of mass spectrometry TIC of different QC samples. Combined FC of metabolite difference and the variable importance in projection (VIP) value of the orthogonal partial least squares discriminant analysis (OPLS-DA) were used to screen DAMs. The FC ≥ 2 or FC ≤ 0.5, and VIP ≥ 1 were considered as the standard of screening. KEGG pathway database was used to annotate the DAMs.

### Extraction and quantification of free amino acids

An automatic amino acid analyzer (Hitachi L-8900, Tokyo, Japan) was used for the determination of free amino acids. The samples were diluted with 10 mL of boiling distilled water and incubated in a water bath at 95°C for 10 min. The extraction was repeated three times and the supernatant was obtained after centrifugation. After adding 10% sulfosalicylic acid, the mixture was centrifuged at 13400 g for 10 min, then dissolved in 0.02 M HCl, and passed through a 0.22-µm pore-size filter membrane into a brown sample bottle for further testing. An autoanalyzer based on the ninhydrin method was used to quantify the free amino acids according to the method described by Ye et al. (2022) (Ye et al., 2022). The amino acid content in each sample was calculated by comparing the peak area of the detected amino acid with that of the standard. All determinations were carried out in triplicate.

### Chlorophyll fluorescence analysis

Chlorophyll fluorescence parameters were measured at room temperature using a pulse-amplitude-modulated (PAM) fluorometer (Imaging-PAM M-series, Walz, Germany). The expanded leaves were dark-adapted for 20 min before measurement. Six different areas of interest (AOI) were randomly selected on each leaf. The maximal quantum yield of photosystem II (*F_v/_F_m_*) was calculated as (F_m_ – F_o_)/F_m_, where F_m_ is the maximum chlorophyll fluorescence, measured immediately after a saturating light pulse, while F_o_, the steady-state chlorophyll fluorescence, was measured after dark acclimation before a saturating light pulse. The effective PS II quantum yield of the illuminated samples was calculated using the formula Y(II) = (F_m_’-F)/F_m_’. The trend of Y(II) and the chloroplast electron transport rate (ETR) parameter was determined on the basis of a rapid light curve (RLC) at PAR of 0, 10, 35, 80, 145, 230, 335, 460 and 610 µmol photons m^−2^ s^−1^.

### Construction and curation of the tobacco genome-scale metabolic model

#### Construction of a draft model

An initial reaction set for tobacco (*Nicotiana tabacum*) metabolism was obtained from Ntabacum_Tn90Cyc and KEGG (Kanehisa and Goto, 2000). Some spontaneous as well as non-gene-associated reactions, including metabolite transport, were also incorporated into the model. A total of 2,116 reactions were extracted from these databases, along with genes, pathways, and empirical formulas of most metabolites as our initial raw data set. To model the inputs and outputs of the metabolic system, 22 extracellular exchange reactions and 47 biomass drain reactions were respectively defined using ‘_Cyto_tx’ and ‘_biomass’ as the subscripts. Extracellular exchange reactions include the import of various energy sources (Glc, Suc, starch, and photons) and mineral nutrients (e.g. NO_3_^-^, NH_4_^+^, SO_4_^2-^, Pi, Ca, Fe, Mg, K) as inputs along with the exchange of CO_2_, oxygen, and water with the environment.

#### Subcellular compartmentalization

The inclusion of gene–protein-reaction (GPR) associations was utilized to localize the individual reactions in the draft model to appropriate subcellular compartments by querying tobacco gene sequences against UniProt database. If it was not available in UniProt, the compartmental information from the GEM (Yuan et al., 2016) of a phylogenetically closely related species, tomato, was used to predict putative cellular compartment for the same reaction. If none of the case above, the WoLF PSORT (Horton et al., 2007) localization prediction software was applied to predict the reaction compartmentalization. Five subcellular compartments, namely cytosol (_c), mitochondria (_m), plastid (_p), peroxisome (_x), and vacuole (_v), are included in the tobacco model, whereby respective suffixes were used at the end of the reaction and metabolite identifiers to distinguish one compartment from another. The cytosol is the default compartment, and any metabolic exchanges between compartments went through the cytosol using intracellular exchange reactions between the cytosol and other compartments denoted with the subscripts _pc, _mc, _xc, and _vc for plastidic, mitochondrial, peroxisomal, and vacuolar exchanges, respectively. The information on intracellular transporters and the energy costs associated with transport was obtained from a published *Arabidopsis* genome-scale metabolic model that has undergone extensive curation (Cheung et al., 2013).

#### Model curation

In order to obtain accurate predictions of metabolic fluxes, genome-scale metabolic models should be curated. Firstly, we revised the directionality and reversibility of reactions based on an *Arabidopsis* (*Arabidopsis thaliana*) genome-scale metabolic model (Cheung et al., 2013) and the defined reversibility of the reactions (Ma and Zeng, 2003). After that, errors of infinite reducing power/energy production were excluded. The protons pumped out from electron transfer reactions of respiratory chains were compartmentalized to the mitochondrial membrane and the proper P/O ratio was guaranteed (the mitochondrial NADH and succinate ratios were 1.875 and 1.125 respectively). Finally, gaps (missing reactions) were identified in biomass components and the biosynthesis pathways of 20 common products (including pyruvate, oxaloacetate and glycerone phosphate) and were filled according to the methods of Luo et al. (2022) (Luo et al., 2022). Generally, the weight-added pFBA gap-filling algorithm was used to introduce the minimal number of reactions from MetaCyc into the model. Each biomass component or common product synthesis pathway was manually curated to make sure its production was less than its theoretical maximum yield, which was calculated according to the ratio of the reduction degrees of substrate to product. Finally, the molar mass of biomass in the biomass production reaction should be 1 g/mmol, and deviations from this value will result in errors in the calculated specific growth rate. Using the BiomassMW algorithm (Chan et al., 2017), we examined the biomass equation of the model and corrected the coefficients of all components.

#### Model validation

The model was checked via constraint‐based flux balance analysis (FBA) (Orth et al., 2010) using the COBRA Toolbox (Heirendt et al., 2019). The ability of iJTC6240 to predict growth rates of transgenic tobacco lines with different photosynthetic capacity grown under field conditions (Jiang et al., 2018) was investigated. In each transgenic line condition, photon influxes were allowed from the photon input reaction with active photosynthesis and with all organic sources of energy set to zero. As most plants uptake nitrogen in the form of nitrate and ammonium, nitrogen input was set to be 50% nitrate and 50% ammonium for the simulation. The rate of CO_2_ fixed was constrained according to the photosynthetic capacity of the respective experimental transgenic lines with biomass maximization as the objective function. The linear programming was mathematically represented as follows:


$$\begin{array}{c} \sum_{j=1}^{n}S_{ij}V_{j}=0\\ v_{j,\min}\leq v_{j}\leq v_{j,\max}\\ V_{m}=t \end{array}$$

where S_ij_ is the stoichiometric coefficient of metabolite i in reaction j, n is the set of all reactions in the model, V is a vector of reaction flux, m is the transporter reaction of each biomass component, and t is the corresponding transporter flux of the biomass component.

### Incorporation of transcriptomic and free amino acid measurement data into the metabolic model

The E‐Flux algorithm (Colijn et al., 2009) was used to contextualize the model by integrating the corresponding transcriptome data of *in-vitro*/soil-grown plants. E‐Flux utilizes gene expression data to set the upper and lower bounds of reactions using gene–protein–reaction (GPR) associations from the model. In detail, the transcriptome data were transformed by log_2_(FPKM+1) using the mean values of three duplicates. The mathematical operations utilized to calculate the numerical values were the sum of “OR” expressions. Finally, the GPR rule values were normalized, divided by the maximum value in each condition. The normalized values were used to establish the new lower and upper reaction bounds. Exchange reactions corresponding to each growth environment were set as −1/1. After that, the condition‐specific models were obtained with specific reaction bounds.

The free amino acid data was incorporated into the model as the biomass component. Under soil-grown condition, photon influxes were allowed from the photon input reaction with active photosynthesis and with all organic sources of energy set to zero. The *in-vitro* condition was modeled by allowing photon influx with active photosynthesis and sucrose as the carbon and energy input (Sucrose_Cyto_tx), with all other constraints the same as the soil-grown one. Constraint-based FBA was run with the objective function of biomass maximization.

### Statistical analysis

For quantitative analysis, values were expressed as the means ± standard errors of three independent biological replicates. Statistical significance analysis was performed using a Student’s *t*-test in Microsoft Excel. A value of *p*< 0.05 was statistically significant.

## Data availability statement

The original contributions presented in the study are publicly available. This data can be found here: https://www.ncbi.nlm.nih.gov/bioproject, PRJNA1014250; https://www.ebi.ac.uk/metabolights, MTBLS8557.

## Author contributions

JY: Formal Analysis, Writing – original draft, Writing – review & editing. XW: Formal Analysis, Writing – original draft, Writing – review & editing. QY: Formal Analysis, Writing – original draft. JS: Formal Analysis. JC: Formal Analysis. ZL: Conceptualization, Writing – original draft, Writing – review & editing, Supervision, Funding acquisition. HM: Conceptualization, Writing – review & editing, Supervision, Funding acquisition.

## Supplementary material

Supplementary Data Sheet 1 Genome-scale metabolic model of iJTC6240 in SBML format.

Supplementary Data Sheet 2 Genome-scale metabolic model of iJTC6240 in Microsoft Excel format.
