# Supplementary material for: Elucidating the impact of in vitro cultivation on Nicotiana tabacum metabolism through combined in silico modeling and multiomics analysis
Source: Front Plant Sci. 2023 Nov 3;14:1281348. doi: 10.3389/fpls.2023.1281348 (PMC10655011; doi:10.3389/fpls.2023.1281348)
Supplement: Supplementary Data Sheet 2 — Genome-scale metabolic model of iJTC6240 in Microsoft Excel format. [file DataSheet_1.docx]

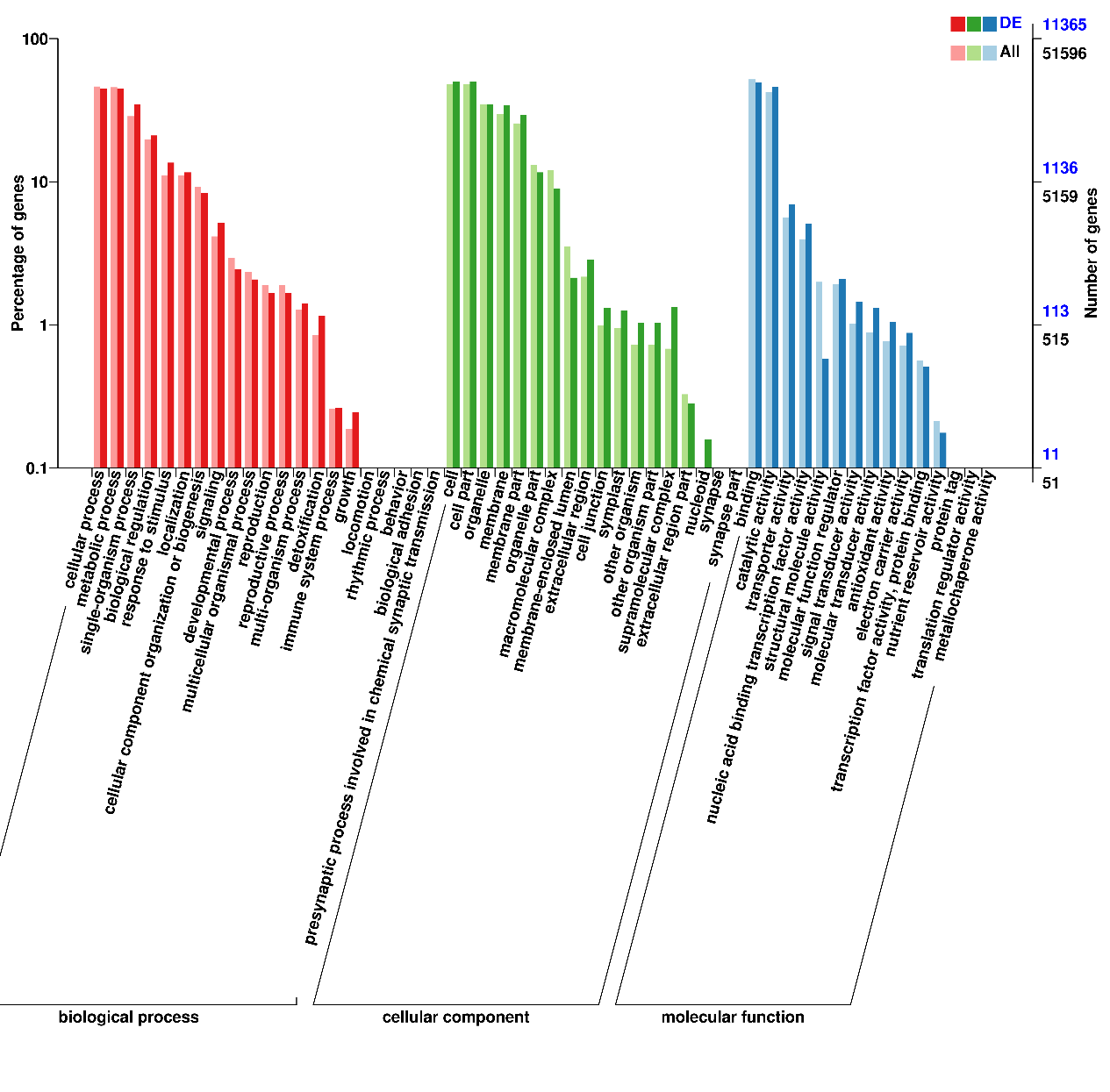


**Figure S1.** **Gene ontology (GO) classification of transcripts of in-soil and in-vitro sets**. The three main GO categories include biological process, cellular component, and molecular function.


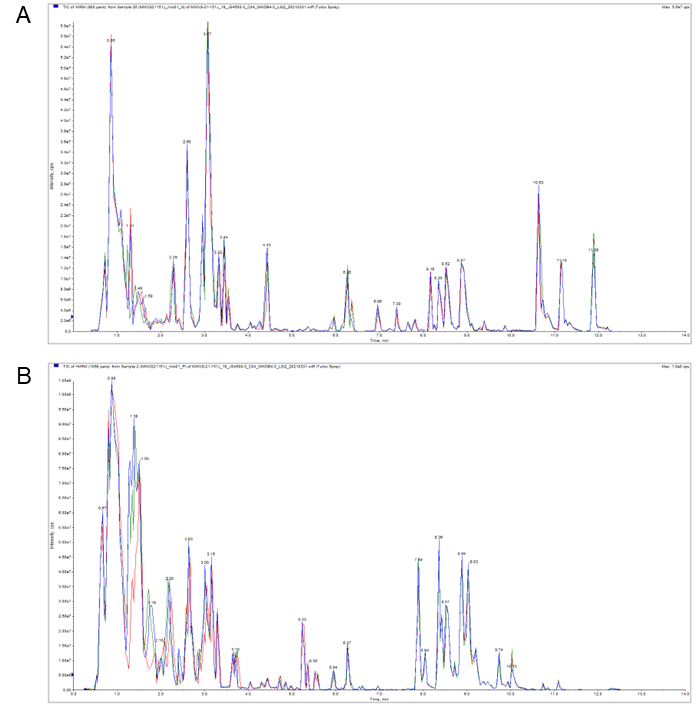


**Figure S2.** **The** T**IC overlap graph detected by essential spectrum of QC samples. A** negative ion mode. **B** positive ion mode. The X-axis is the retention time (RT) of metabolite detection, and the Y-axis is the ion current intensity (the intensity unit is CPS, count per second).


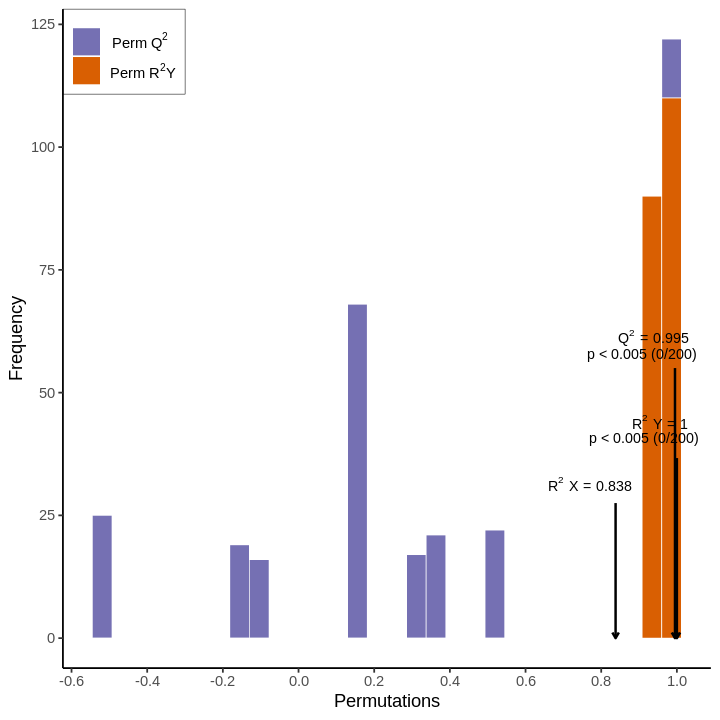
 **Figure S3.** Orthogonal Partial Least Squares Discriminant Analysis (OPLS-DA) model permutation test.


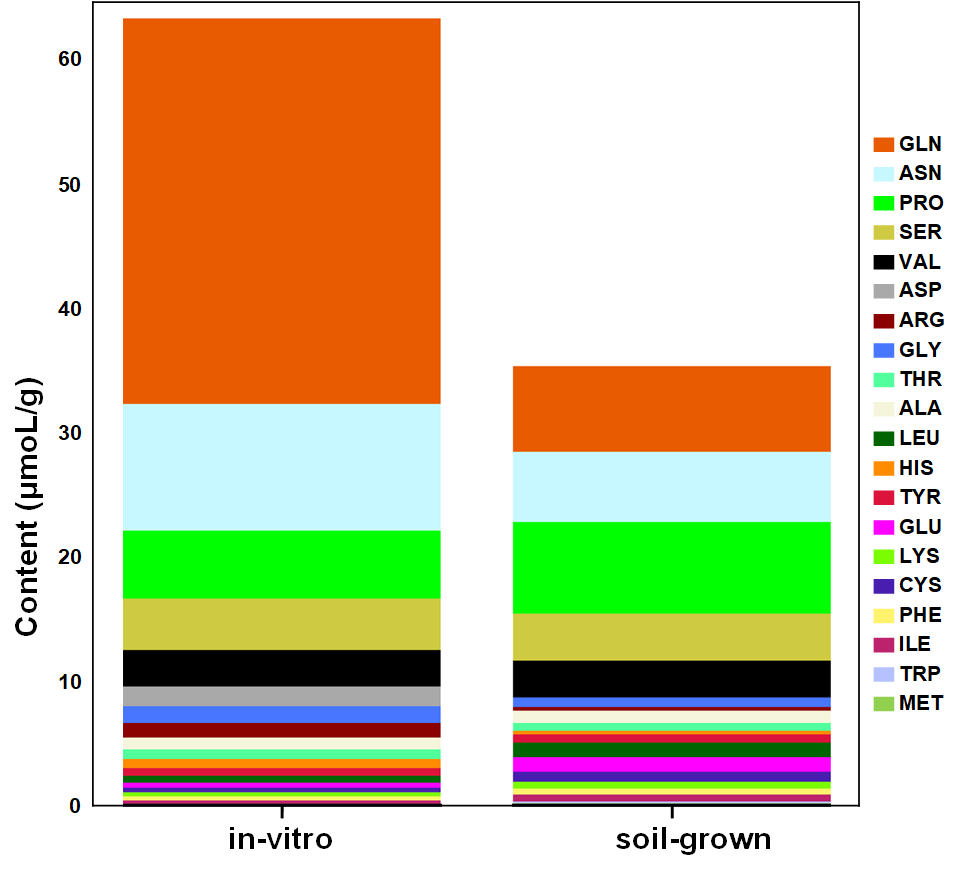


**Figure S4.** Changes in the absolute concentrations of common amino acids in 7-WAS tobacco between in-vitro and soil-grown conditions.
